# Skimming stones

**DOI:** 10.1093/sleepadvances/zpad026

**Published:** 2023-08-29

**Authors:** John Marshall Orem

**Affiliations:** Texas Tech University Health Science Center, Lubbock, TX, USA

**Keywords:** sleep/wake physiology, animal models, basic science, control of breathing, REM sleep, REM sleep behavior disorder

Look back on time with kindly eyes,

He doubtless did his best;

How softly sinks his trembling sun

In human nature’s west!

Emily Dickinson

## A Career by Chance

Dr. John (Jack) M. Rhodes’ laboratory was a WWII barracks among adobe buildings at the Albuquerque campus of the University of New Mexico. In the spring of 1966, The Albuquerque Journal published a photograph of me, my wife, and our young daughter as part of a story on inductees into Phi Beta Kappa. Dr. Rhodes saw the story and invited me to his laboratory. He asked about my plans and, learning I had none, invited me to work in his lab. That was the beginning of my career. A beginning that happened just by chance.

He provided resources for students, even a computer in 1968, and if he did not know something he brought in someone who did. But he had charm and charisma. Tall and good looking he handled his cigarette like an ornament. Not surprisingly he was a local thespian. The laboratory saw ladies come and go as patients under his spell. And he had collaborations with French scientists. I was asked to tour Professor Fessard of Paris who was known for showing that the brain was spontaneously active in the absence of stimulation. This was apparently unexpected of a reflexive brain. His students in physiological psychology, it was before the era of “neuroscience,” benefited from his allure in a department of strict behaviorists.

Allan Netick was the senior graduate student in his lab. They had a special relationship. Allan was to the manor born as Jack seemed to be. Or perhaps there was just a good understanding. I knew only that Allan insisted that Jack stay out of the lab when we were experimenting. It was a mandate Jack willingly accepted. Jack’s science had to do with electroencephalography, which out of some arrogance we did not respect. Our research we imagined was more substantial. Jack the provider brought in Henri Korn to teach me how to record single neurons.

Allan was a graduate of Reed College, did the crossword puzzle mornings, was brilliantly cynical, and highly intelligent. Ed Bixler also a student of Jack’s helped me with technical matters. He was a handsome track star, and an aficionado of motorcycles and cars. He bought a Ducati motorcycle and the controls were the reverse of those on his previous motorcycles which caused him to go over a cliff in the mountains of New Mexico. He spent many weeks in the hospital with only a brick wall to see out his window. I sneaked in Bloody Marys for some relief.

When I was a young professor, Jack, near the end of his life, came to Texas under the pretense of a business trip, but the real business was just to see me.

## UCLA and Stanford

In 1970 I took a postdoctoral fellowship with Dr. John Schlag at the Brain Research Institute at UCLA. I borrowed my father’s pickup and left my wife and children in an empty house in Albuquerque while I moved our things to Los Angeles. I arrived late at night, found Ed Bixler’s home and thinking it too late to wake them I went to sleep on their lawn. I was awakened by more than one policeman, colored lights flashing all around. Welcome to Los Angeles.

Working with Dr. Schlag meant working every day all day in the same room with him and his keen mind. He was not the mentor of an organization of postdocs. It was just the two of us. He puzzled over the function of fragments of surplus equipment and asked, for example, how we know the difference between movement of our eyes and movement of the world. The retinal pattern could be the same. Those were the days of the Sylmar earthquake, and the question seemed related to the experience of aftershocks on the 8th floor of the Brain Research Institute. I knew it was an aftershock and not a dizzy spell if I saw the cables swinging in the lab. Dr. Schlag believed that study of the functioning brain should be in an intact un-anesthetized animal. I built apparatus for that to study the role of the thalamic internal medullary lamina in eye movements.

Dr. W.C. Dement at Stanford contacted Dr. Schlag inquiring about candidates for a postdoctoral fellowship. Dr. Dement was perhaps the most important leader of the study of sleep and sleep disorders in the 1970s. It was the beginning of sleep disorders medicine fueled largely by recognition of obstructive sleep apnea, which notwithstanding Charles Dicken’s description of Joe the fat boy in *The Pickwick Papers,* can be attributed to Gastaut, Tassinari and Duron who published in French (1965) and in English (1966) [1] a thorough description of the syndrome. But it was Dement’s organization at Stanford that took up that and other sleep disorders and led the field. I was lucky to be there from 1972 to 1976. My studies there with Jacque Montplaisir and W.C. Dement showed respiratory neurons whose activity decreased in sleep [2] and with Allan Netick an increase in upper airway resistance in sleep [3] and medullary neurons active only in REM sleep that coded both tonic and phasic features of that state [4]. Subsequent studies by others indicated that those medullary cells were involved in the atonia of REM sleep.

But major contributions can come from unexpected sources. At Stanford every year we submitted abstracts on work to be presented at an annual international meeting. One year my abstract was about the above medullary neurons. That same year, Jerry Holland, on sabbatical at Stanford, submitted an abstract proposing that the term “polysomnography” be adopted to refer to the procedures used to evaluate patients with sleep complaints. I thought this ludicrous not only because of the mixing of Greek and Latin roots (as Michel Jouvet noted), but because the abstract had no scientific substance. However, the word was needed. Instead of nebulous statements about electrodes being placed here and there to record this and that along with a host of respiratory parameters it could be written, medically sounding, that the patient was evaluated using polysomnography. The insurance companies liked it and paid, and the discipline of sleep medicine flourished.

## Physiology in Sleep

I left Stanford in 1976 and came to Texas Tech Medical School’s Department of Physiology. At the suggestion of Bill Orr, and with encouragement from Pier Luigi Parmeggiani, I, with Charles Barnes, edited the book Physiology in Sleep [5]. The contributors were from various fields of physiology who notably did not know each other. Their colleagues were in their respective fields (eg, cardiovascular, endocrine, or respiratory physiology) and not in the area of sleep research. The book became required reading in the new field of sleep disorders.

In 1978, Ralph Lydic and I found what we thought was the key to understanding why the airway collapses in sleep apnea [6]. We wrote: “*The finding pertinent to the obstructive sleep apneas is the preferential excitation of the laryngeal abductors by the activating system of Moruzzi and Magoun.”* Preferential because the activating system excited airway dilator muscles more than abdominal and chest muscles that create the negative pressure of breathing. We believed that the other side of this preferential excitation was a possible preferential inactivation of the airway dilators when reticular tone decreases. The ability to dilate the upper airways on arousal becomes the liability of occlusive collapse on the loss of wakefulness.

Our findings were confirmed by a long line of studies by Walter St. John at Dartmouth all of which were published without acknowledgement of our priority [7]. Eventually after it was said and done I wrote to Walter and asked why he had not cited us. (Such is our pride.) He responded apologetically that he was not aware of our study and that we were indeed the first to publish what he eventually came to understand.

In the 1970s sleep researchers, but not general practitioners, were aware of obstructive sleep apnea. I attended a yoga class in Palo Alto in the mid 1970s, and a fellow next to me invariably fell asleep and became obstructed during relaxation at the end of the sessions. Knowing that there was little chance that his physician would know what to make of his symptoms, I decided to talk to him. And so one evening I said to him that he had a problem, and I knew what it was. To which he responded, “Mind your own business.”

## To Be or Not to Be a Respiratory Neuron

In 1981 Allan Netick and I published a paper entitled *Erroneous Classification of Neuronal Activity by the Respiratory Modulation Index* [8]. Andre Hugelin’s group in Paris used this RMI to map respiratory activity in the brain [9]. They found with it that the reticular activating system of the midbrain, an area important for wakefulness, contained many respiratory neurons, that is, neurons whose activity was related to some phase of the breathing cycle. I thought this activity could explain the important wakefulness stimulus to breathing that when lost in sleep leads to apnea. And so I set out to record the cells in the reticular activating system. However, none of my recordings of some 800 neurons in that area had activity related to breathing. Hugelin had published that 30% of the cells there were “respiratory.” If this were so the probability of my not finding one with respiratory activity (*p* = 0.7) in 800 attempts would be an infinitesimal 0.7^800^. I programmed a Southwest Technical Products 6800 microprocessor to analyze random “neuronal” activity using Hugelin’s formulae and found that the respiratory modulation index (RMI) often erroneously classified the random activity as having a relation to the respiratory cycle.

I was leaving for a month in New Zealand and Australia (with Christian Guilleminault), and, worried about the long flights on DC-10s whose engines on rare occasions had separated from the wing, I packed up the results of my simulations and sent them to Allan in California. While I was away Allan, using then high-powered computers at California State University, proved that the RMI was a flawed statistic. It produced both false positive and false negative errors. Non-respiratory activity was deemed respiratory and respiratory activity was, in some cases at a high rate, deemed non-respiratory. Allan showed also that the problem of detecting respiratory activity could be solved using an analysis of variance (Anova) in which fractions of the respiratory cycle was the treatment variable and successive breaths the subject variable. He showed that Anova was a powerful technique for detecting even the slightest respiratory modulation in the activity of a cell.

In April of 1982 Allan and I confronted Hugelin’s colleagues at a meeting in Lake Bluff, Illinois. The high pitch of my voice when I presented our results amused Allan. I remember my nervousness about possibly erroneously claiming that they were wrong. But we were right.

Reactions to our work varied. Some said it was wrong because Hugelin was a respected scientist, a member of the prestigious Centre Nationale de Recherche Scientifique; others that it was right because of his putatively flawed character. A senior French scientist, Dr. Dussardier, wrote to me saying an examining committee had failed one of Hugelin’s students whose work depended on the RMI and who had no response to our paper. I was relieved to read his assurance that the student, who was not at fault in creation of the flawed statistic, would receive eventually the doctoral degree.

It was the end of Hugelin’s program that had produced erroneous three-dimensional maps of respiratory activity in the brain, and it was the end of his career on the neural control of breathing, a career that in his early work included the important discovery that stimulation of the reticular activating system causes excitation of respiration. The literature contains their results and our refutation [8, 9], both now having only historical interest. The stories of science are sequences of positive results. Their findings and our refutation of them have entered the domain of the un-cited.

In 1983 on a sunny July day in Paris my wife Elizabeth and I had lunch with Andre Hugelin. After lunch we stood on the walk to say goodbye. I was facing west with the sun on the left side of my face. Hugelin was behind me and to my right, and I turned to face him and say goodbye when he said, “You know that I am right.” I hesitated then said, “Yes.”

The Significance of Respiratory Effect Size

The story did not end with proof that the RMI was a faulty statistic and that Anova powerfully detected relations between breathing and neuronal activity. Results of the Anovas (F-ratios) varied widely among respiratory neurons. At the lowest end of the range of significant F-ratios the relation of the activity of the cell to breathing was so weak that Anova was required to detect it, whereas at the upper end the relation was obvious without any analysis. Anova allowing only rejection of the associated null hypothesis of no difference in activity across fractions of the respiratory cycle was clearly an inadequate quantification of the quality of “respiratoriness” of the cells. I was musing about this to a friend who suggested that what we needed was an effect size statistic. And accordingly Ted Dick and I showed that an effect size statistic, η^2^, could be used to quantify “respiratoriness” [10]. We applied it to a group of respiratory neurons and showed that the η^2^ statistic describes properties of the activity of a cell that are constant within a state. We did not know what this meant as a completion of a statement such as: “For the respiratory system *to do such and such* then there must be a family of respiratory neurons showing a spectrum of η^2^ values.”

Two years later, with co-authors Ivan Osorio, Ted Dick and Edward Brooks, I published a paper entitled *Activity of Respiratory Neurons in NREM Sleep* [11]. In the introduction is written: “*the physiological significance of the η*^*2*^*statistic, other than a quantitative description of the respiratory component in the activity of a cell, is unknown, but, in the experiments described in this paper, we have found a relationship between the η*^*2*^*value of the activity of a respiratory cell and the size of the effect of sleep on that cell.”* This relationship was negative: the higher the η^2^ value the less the reduction in discharge rate in sleep. Some low η^2^ value cells ceased discharging altogether in sleep whereas high η^2^ value cells discharged the same number of action potentials per breath in sleep as in wakefulness but at a lower discharge rate. (Indeed there was a strong inverse relation between discharge rate and the duration of the breath for high η^2^ value cells.) We concluded that, assuming that the η^2^ value of activity is related inversely to the amount of non-respiratory input contributing to that activity, this indicates that the effect of sleep is greater on cells receiving larger proportions of non-respiratory input. The vulnerability of breathing in sleep was we thought the result of this effect, because it meant that structures subserving multiple roles only one of which was respiratory would be at greater risk during sleep.

A year later Allan and I published a paper entitled *Behavioral Control of Breathing in the Cat* [12]. Previous studies claimed that behavioral control occurred in the spinal cord where motoneurons cause contraction of respiratory muscles, but we showed it occurred in the brainstem where the respiratory rhythm is generated. Cats were trained to stop inspiration when a conditioning stimulus (CS) sounded at the onset of the inspiration. When they performed this response, brainstem neurons that normally discharged during inspiration stopped. I wondered what stopped them. There were several cell-types known to inhibit inspiratory neurons^,^ but I found that none were activated during the response. Therefore, they were not the cause of it. Indeed their inactivity during the response indicated that they were a part of the response and not the cause of it. My thoughts about this were slow to develop. I was fixated on the inhibition of inspiratory neurons and slow to recognize that the brain’s response to the task was to stop not just inspiratory neurons but all components of the respiratory oscillator including those that in the course of automatic breathing inhibit inspiratory neurons.

The inspiratory cells that were inactivated behaviorally, and those cells that I thought might cause this inactivation, had high η^2^ values. Their periods of activity and inactivity occurred precisely at given phases of the respiratory cycle and were consistent from breath to breath. This suggests that they were the elements of the oscillator itself^.^ Other respiratory cells became very active at a short latency in response to the task. They had the following characteristics [13]: (1) As a group, they had discharge profiles related to every phase of the respiratory cycle; (2) They were recorded in the same region as, and often simultaneously with, respiratory cells that were inactivated; (3) The η^2^ value of their activity was low; (4) The latency of their activation was significantly shorter than the latency of inactivation of high η^2^ valued inspiratory cells; (5) This activation was intense and prolonged.

I published these results in 1989 [13] and theorized that the low η^2^-valued cells were the elements that stopped inspiration during the task. It made sense that the brain contained cells that were not constrained by rigid sequences of postsynaptic potentials and were the interface between the constrained oscillator and inputs involving behavioral and reflexive control of it. With these ideas physiological sense was made of statistical results.

The Question of Using Animals for Research

That year, 1989, the Animal Liberation Front (ALF) broke into my laboratory, destroyed equipment, and “liberated” our experimental animals. With news of the crime People for the Ethical Treatment of Animals (PETA) took up the justification of it in a propaganda war that would go on for more than 2 years.

Here is how they worked it. The crime was committed to get the attention of the press who then published PETA’s message that the break-in was justified by my cruelty in the treatment of the experimental animals (cats). And what is more interesting to the press? Damaged equipment or a cruel scientist, a “*Mengele f..ker”* according to one who wrote to me? Furthermore perceived authorities (physicians affiliated with PETA) claimed my work had no redeeming value. So what is more newsworthy? A physician’s finding of worthlessness or my attempt to defend basic research as perhaps someday useful? The accusations against me, the victim, triggered investigations by the university whose committees had approved my research, the National Institutes of Health who had funded it, and by the local, state, and national press. Results showing the absence of fault came long after the accusations and were less newsworthy. I received more than 10 000 pieces of protest mail, some containing death threats. Police trained me to use a gun.

I met with an ad hoc committee without which it would have been a lonely experience. Two members wrote a book (*Targeted. The Anatomy of an Animal Rights Attack*. by Lorenz Otto Lutherer and Margaret Sheffield Simon [14]) that described in detail the attack. A third member (James E. Heavner, D.V.M., Ph.D.) provided structure and order to the meetings without which they would have been pointless. A fourth member (Peter A. Doris. Ph.D.) played the activists’ game with mischief and humor. As written in *Targeted,* the attack was directed at me and not on the worth of biological research.

These events triggered many more. Here are some that happened then: trips to Washington D.C. to attend events for support of animal research and to be interviewed by a national talk show host; Meeting Senator Lloyd Bentsen and Dr. Michael DeBakey who spoke in support of me and animal research; The interview on national TV that my mother on the Montana ranch just happened to see; A meeting in San Antonio where I invited an activist to come to see what I actually did (She declined.); Billboards throughout Texas showing one of my experimental animals with a recording headcap and who had a Horner’s syndrome (unequal pupils); An interview with a reporter from the Texas Monthly whose photographer took photo after photo until I showed disgust, and he got then the picture that he wanted all along; Grief at news of an investigation of me by the administration of the university; The great actor Richard Kiley filmed narrating a piece featuring PETA’s case against me entitled “No Gravy for the Cat”; A meeting with a lawyer who wanted to sue PETA using the RICO act and who, when I declined to participate, said scientists, having developed heart and kidney transplants, needed to work on a backbone transplant; In May 1998 at Copan in northern Honduras, I was approached by someone who asked if I was John Orem. I was shocked wondering what would happen next until he explained he was the editor of the book *Targeted* and recognized me from it.

PETA wanted to keep the case alive, and that required my participation. After 2 years I walked away, and the case died down.

## Photographs of Respiratory Neuronal Activity in an Intact Cat During Normal Sleep and Wakefulness and During Behavioral Control of Breathing

**Figure 1.**
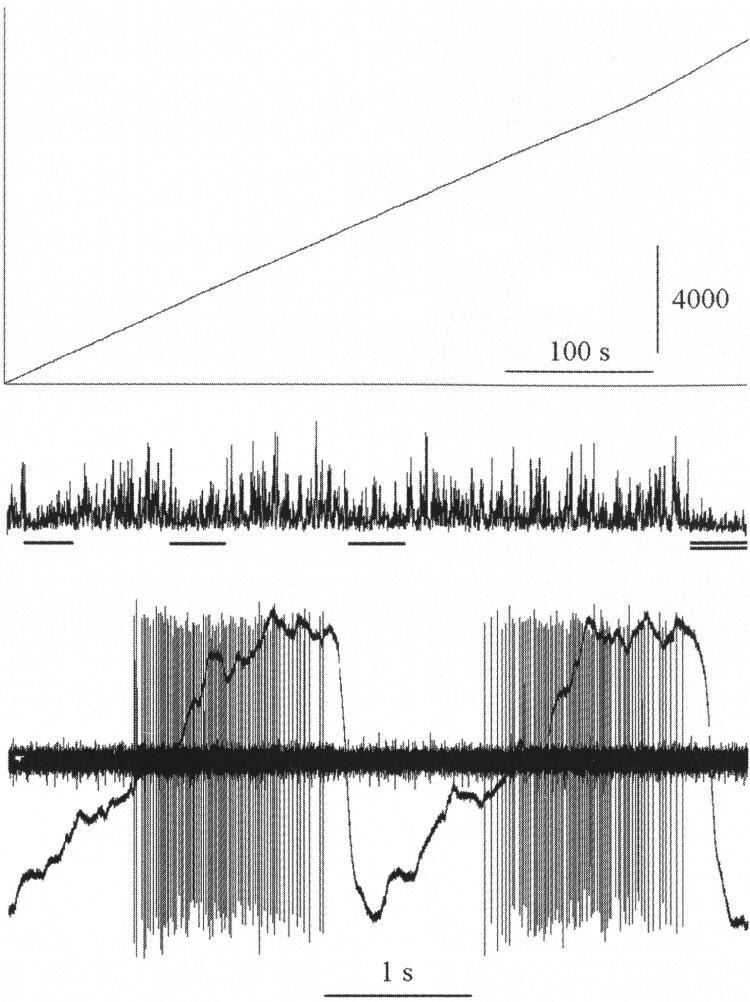
Activity of a single respiratory neuron. The lower section shows the action potentials of the cell during two breaths. The trace overlapping the action potentials shows breathing with an upward deflection signifying inspiration. The upper section shows the cumulative action potential count of this cell. The unvarying slope of the count during NREM sleep and wakefulness (denoted by a single underbar) indicates that the activity of the cell was constant during these states. The upward deflection of the count at the end of the record when REM sleep occurs (double underbar) indicates an increase in activity in that state. The trace above the underbars is the half-wave rectified electroencephalogram (with permission from ref. 15).

In Figure 1 the lower part shows action potentials of a neuron during two breaths. From left to right is a time period of only about 5 s during which two breaths occur. Two blocks of fine vertical lines (one block for each breath) show the action potentials of one of the approximately 250 million neurons (!) in this animal’s brain. Each action potential has a duration of about one-thousandth of a second. There are approximately 50 action potentials each second during the active period of the neuron, an active period that begins at the end of expiration and continues almost to the end of inspiration. Expiration is shown as a downward deflection of the continuous curve that overlaps the tracings of the action potentials. Upward deflection of the curve signals inspiration. This neuron would be named an “expiratory-inspiratory” neuron because of the time in the respiratory cycle when it is active. It discharges at a rate of about 50/s and its pattern of discharge is consistent from one breath to the next and therefore has a high η^2^ value. We expect that the activity of the cell in Figure 1 will continue stably in NREM sleep but will increase significantly in REM sleep (see below).

In the upper part of Figure 1 the lower tracing shows the half-wave rectified electroencephalogram with arousal to wakefulness denoted by single bar underscores and REM sleep denoted by the double bar underscore at the end of the record. The diagonal line in the upper part shows the cumulative action potential count of the neuronal activity of the cell over a period of about 9.4 min. The slope of the diagonal line is constant throughout this period of sleep with several arousals then steepens in REM sleep. This indicates a constant rate of action potentials in wakefulness and NREM sleep and an increased rate in REM sleep [15].

**Figure 2.**
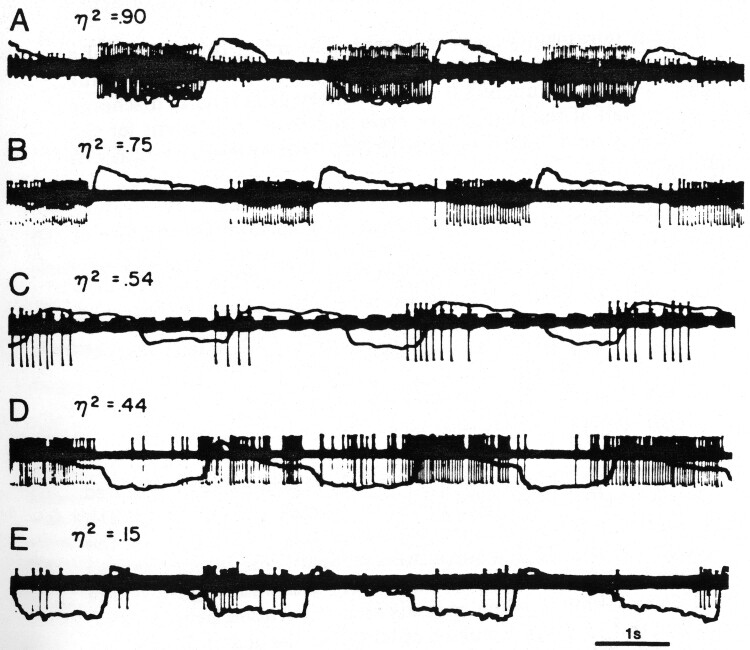
Respiratory neurons (A–E) having different η^2^ valued activity. Action potentials are superimposed on the breathing trace with inspiration signified by a downward deflection (with permission from ref. 8).

**Figure 3.**
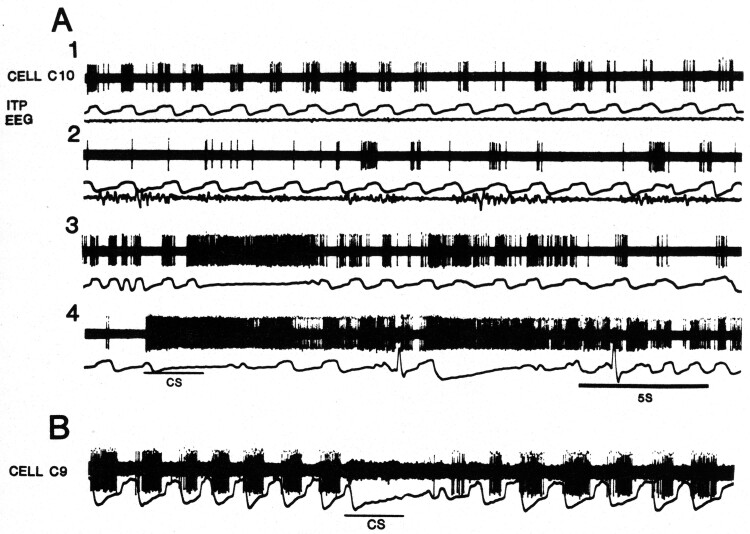
(A) Activity of an inspiratory neuron (top tracings in 1–4) during wakefulness (A1), NREM sleep (A2), a spontaneous apnea (A3), and apnea in response to a conditioning stimulus (CS) (A4). In A1 and A2 traces are from top down: action potentials of cell C10, intra-tracheal pressure (ITP), and electroencephalogram (EEG). In A3 and A4 only the action potentials and ITP are shown. (B) Activity of an expiratory neuron during breathing and inactivity of the cell during behavioral inhibition of breathing (with permission from ref. 11).

Figure 2 shows examples of respiratory activity having different η^2^ values. The η^2^ statistic is an effect size statistic that can vary from 0 to 1, indicating respectively no relation to breathing to a relationship in which all the variability in activity is accounted for by its relationship to the respiratory cycle. The η^2^ value is the proportion of total variance of the neuronal activity over a series of breaths that is made up by the variance across fractions of the respiratory cycle. There are few speculations about the functional significance of this spectrum. Some experiments indicate that high η^2^ valued cells belong to an oscillator that drives breathing and that low η^2^ valued cells act as an interface for behavioral control of the oscillator (Figure 3). The latter are intensely activated when breathing is stopped behaviorally, and as a class they are less active in sleep. Figure 3A shows this: trace 1 shows inspiratory activity in wakefulness that in sleep (trace 2) is irregular. The cell is intensely active during a spontaneous apnea (trace 3) and during behavioral inhibition of breathing elicited by the CS (trace 4).

These data were obtained from cats studied over a period of many weeks during which they were named, showered with good husbandry by veterinarians, and an employee whose only job in the laboratory was their care. They were un-drugged and healthy during the study. The experiments were sanctioned by the Institutional Animal Care and Use Committee of Texas Tech University School of Medicine and by the National Institute of Health that funded them. That was over 30 years ago. The climate has changed now. Rats, mice, and fruit flies are the subjects of choice, and they do not lend themselves to experiments like those shown in the figures here.

**Figure 4.**
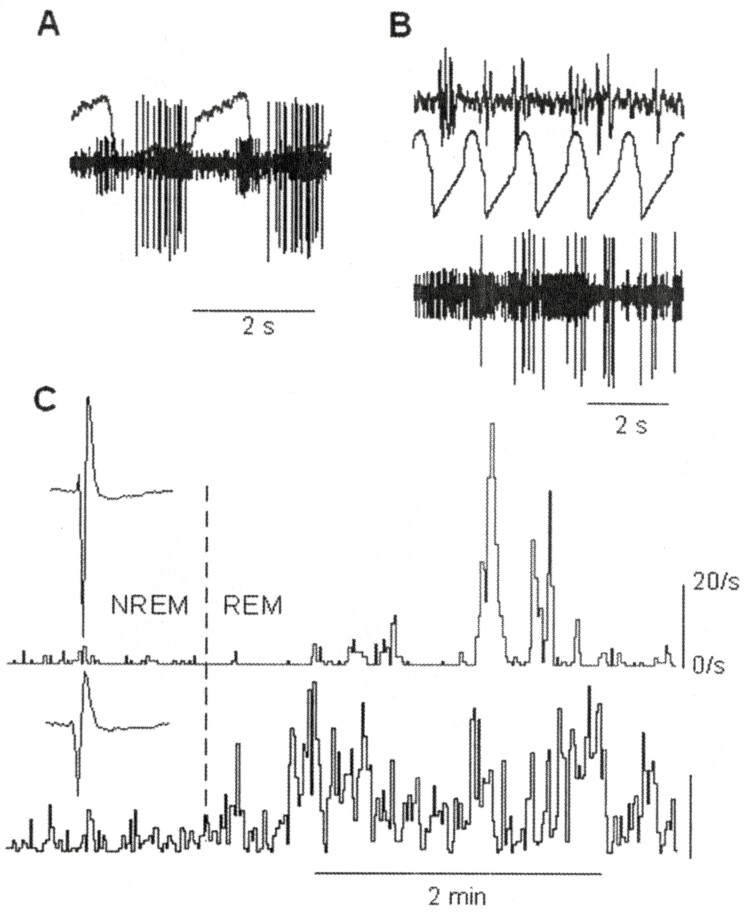
(A) Activity of two respiratory neurons recorded simultaneously during two breaths. Intra-tracheal pressure overlaps the action potentials (inspiration signified by an upward deflection). (B) Bottom trace: activity of the two cells during ventilator-induced apnea in REM sleep. Top trace: ponto-geniculo-occipital waves (signifying REM sleep). Middle trace: intra-tracheal pressures created by the ventilator to produce apnea (upward deflection signifies inflation). (C) Activity of the two cells during ventilator induced apnea in NREM and REM sleep. The activity of the cell showing larger action potentials is illustrated in the top tracing. The activity of the cell showing smaller action potentials is illustrated in the bottom tracing. The dashed vertical line denotes the transition from NREM to REM sleep (with permission from ref. 17).

Mechanical ventilation easily stops spontaneous breathing. Dr. Edward H. Vidruk and I tried to determine how it happens that the respiratory oscillator in the brainstem stops when mechanical ventilation is applied, but we were unsuccessful. We realized, however, that we could stop the animals’ breathing with mechanical ventilation during both sleep and wakefulness while at the same time recording the activity of individual respiratory neurons, and that, with this technique, the respiratory component in the activity of the neuron is removed revealing only the non-respiratory components [16]. Figure 4 shows an example of this. In Figure 4A two normal breaths are shown along with the activity of two respiratory neurons—one with large, the other with smaller action potentials. In B the animal is ventilated in REM sleep and although the animal is not breathing both respiratory neurons are active. Their activity does not have a respiratory pattern, and we can only wonder about the meaning of that activity. In C activity during ventilator induced apnea before and during the REM period of the two neurons is plotted. REM sleep begins and after a short delay (about 30 s) something else begins within it, a mysterious excitation that runs through the neurons of the brain causing them to discharge in generally undecipherable patterns. Does the activity shown by the cells in C follow from dreams or what?

## Chairman, Students, and Questions

I was chairman of the Department of Physiology at the Texas Tech Health Science Center for seven years (1997–2004). During that time, and the prior period involving the attack by activists, graduate students Rodney Trotter, Andrew Lovering, and Jimmy Fraigne with the help of Becky Tilton and Edward Vidruk carried on studies largely without me and with interesting results. I appeared in the laboratory often only to take a nap, the later requiring just a few seconds. (Is there an explanation of the restorative effect of so little sleep?)

Rodney Trotter found that augmented breaths occurred often at the transition from wakefulness to sleep. The pattern was a deep inspiration in wakefulness followed by a prolonged expiration during which the cat fell asleep [17]. Augmented breaths have long interested physiologists who know that they promote the spread of surfactant in the lung without which the work of breathing becomes problematic.

It may be that the relation between augmented breaths and state transitions explains why deep breathing is recommended for relaxation. We were not the first to observe this relation. Almost 15 years before us, McGinty et al. [18] wrote that augmented breaths in kittens occurred as part of “*an ordered sequence which frequently included increased somatic activity, a sigh or augmented breath, a co-occurring variable heart rate deceleration and sleep state transition… these phenomena might be caused by some underlying excitatory process which occurs periodically.”*

Andrew Lovering found that the insomnia common at high altitude was not the result of low oxygen concentrations (as John Pappenheimer insisted) but rather low levels of carbon dioxide in the blood that resulted from the response to the low levels of breathed oxygen [19].

Jimmy Fraigne showed with Andrew Lovering that low carbon dioxide concentrations in the blood causes insomnia, but also that moderately elevated levels of carbon dioxide increase sleep [20]. Jimmy described also the time course of the excitatory process that excites and perturbs the respiratory system in REM sleep [16, 21–23].

Irma Aguilar, a hospice nurse, took me on her rounds. She believed that dreams were important for achieving acceptance of one’s death. I asked one patient about her dreams the night before. She said they were bad. In her dream she died, and her children went through her things throwing much away and selling her house. Then she said, “Thank God it was only a dream.”

As fate would have it, Allan Netick in “only” a (violent?) dream would lunge from his bed in his course of REM Sleep Behavior Disorder and Parkinson’s Disease.

## Notes

This paper is part of the Living Legends in Sleep Research series, which is sponsored by Idorsia Pharmaceuticals and Jazz Pharmaceuticals.
